# A dual process for the cognitive control of emotional significance: implications for emotion regulation and disorders of emotion

**DOI:** 10.3389/fnhum.2014.00253

**Published:** 2014-04-25

**Authors:** Steven G. Greening, Tae-Ho Lee, Mara Mather

**Affiliations:** ^1^Davis School of Gerontology, University of Southern CaliforniaLos Angeles, CA, USA; ^2^Department of Psychology, University of Southern CaliforniaLos Angeles, CA, USA; ^3^Neuroscience Graduate Program, University of Southern CaliforniaLos Angeles, CA, USA

**Keywords:** cognitive control, emotion, attention, emotion regulation, depression, prefrontal cortex, arousal

Emotion regulation involves the cognitive control of inappropriate or unwanted emotional reactivity. Modern use of blood oxygenation-level dependent (BOLD) functional magnetic resonance imaging (fMRI) has begun to elucidate the neural regions whose activity correlates with emotion regulation. Research has focused on prefrontal involvement in emotional control, with the dorsolateral aspect (dlPFC) interacting with the ventromedial prefrontal cortex (vmPFC) and amygdala to modulate emotional reactivity (Johnstone et al., 2007; Delgado et al., 2008; Phillips et al., 2008). There has been less focus on how prefrontal control regions might interact with other regions. For example, it is suggested that, consistent with the role of voluntary attention in the biased competition model (Desimone and Duncan, 1995), emotion regulation works by augmenting goal-relevant stimulus or semantic representations in the occipitotemporal cortices (Blair and Mitchell, 2009; Buhle et al., 2013). In this manner, while processing resources are prioritized to goal-relevant representations, unwanted emotional information is indirectly suppressed. The resolution of this debate has been slowed partly due to the low temporal resolution inherent in the BOLD signal. With fMRI it is unclear whether modulation of a particular region occurs following either stimulus onset or regulation onset. A recent article in the Journal of Neuroscience by Harris et al. (2013) reconciles some of the ambiguity regarding the neural underpinnings of emotion regulation. Specifically, their findings suggest a dual process model for the cognitive control of emotional significance.

Harris et al. (2013) overcome the temporal limitations of fMRI by using electroencephalography (EEG), and provide novel insight into the neurocognitive mechanisms involved in the cognitive control of positive stimuli. They measured event-related potentials (ERPs) first while participants viewed food options and reported which they would prefer to eat, and later while they attempted to exert self-control over their selections. In other words, in the critical self-control session participants were required to regulate their emotional response to rewarding food cues in favor of goal-relevant healthy choices. Harris et al. (2013) observed an increase in the N1 amplitude of the occipital cortex between 150 and 200 ms in successful relative to failed self-control trials, which as they note is thought to relate to the attentional modulation of early visual cortices. Importantly, significant occipital modulation did not occur in the natural choice session in which participants were not required to control their responses. The authors also found that successful self-control trials were associated with significantly higher phase-locked activation in the alpha-band between dlPFC and occipital cortex during the 100–150 ms after stimulus onset. This supports the interpretation that the dlPFC was involved in the modulation of occipital activity during the self-control conditions early on in information processing. Thus, it appears that attentional processes involving the dlPFC can modulate affective significance by filtering out unwanted stimulus representations of emotional significance. Such a mechanism resembles perceptual selection and top-down biased-competition (Desimone and Duncan, 1995).

In addition to the early attentional process, Harris and colleagues observed that dlPFC modulates vmPFC activity at a later stage of information processing, which is important for influencing the representation of affective value. Specifically, the vmPFC activity was modulated as a function of self-control and Granger causality analyses indicated dlPFC modulated this vmPFC activity at a later time point than the dlPFC-occipital interactions (400–650 ms). Putting their findings together, Harris et al. (2013) conclude that the self-control of desirable food is a product of both the early attention-related filtering of occipital cortices, and the late modulation of the vmPFC valuation system.

Although Harris et al. (2013) briefly note that their findings might have implications for models of emotion regulation, they do not go far enough in relating their findings to the broader context of emotion regulation. Below we discuss the implications of Harris et al. (2013) findings for models of disorders of emotion with a particular emphasis on the relevance of their early attentional filtering findings to depression. Later we briefly discuss a model which may prove useful in elucidating the precise role of early attention in emotion-related processing, including emotion regulation.

To date, influential theories (Phillips et al., 2008) and empirical studies (Johnstone et al., 2007) regarding emotion regulation impairments in disorders of emotion, including depression, have justifiably focused on the prefrontal cortex—sometimes masking out all other cortical regions—and their subcortical connections. Indeed the findings of Harris et al. (2013) confirm the potential importance of connections between the dlPFC and vmPFC in the modulation of value-based behavioral responding. However, their findings also highlight that those same models have overlooked the importance of dlPFC connections to, and the attentional control of, perceptual cortices involved in early stimulus and semantic representations. This is consistent with novel evidence demonstrating that in individuals with depression there is abnormal modulation of early perceptual regions during emotion regulation (Greening et al., 2013) and that occipital cortex reactivity to emotional stimuli predicts future treatment response (Furey et al., 2013). Together, such findings emphasize that future work is needed to elucidate how emotional significance might interact with attention-related neural mechanisms in early perceptual regions to influence psychopathologies like depression.

The arousal-biased competition (ABC) theory may provide a useful framework for testing predictions regarding the interactions between attention and emotional reactivity. It builds on the bias-competition framework by incorporating emotional arousal. ABC suggests that emotional arousal strengthens and sharpens neural representations of high priority (goal relevant and/or perceptually salient) cues, while suppressing representations of low priority cues in a winner-takes-more and loser-takes-less manner (Mather and Sutherland, 2011). ABC accounts for the seemingly disparate findings that at times emotions can enhance neural activity and facilitate behavior, while at other times they produce impairments (Lee et al., 2014). During emotion regulation, one prediction is that the suppression of unwanted emotional percepts requires increased attentional control during arousing situations. Emotional percepts tend to be intrinsically high-priority. When emotionally aroused, they should dominate processing even more, thus requiring enhanced attention control to be suppressed. In this manner, ABC may serve as a useful model for future research into the role of attention in the early modulation of emotional representations in occipitotemporal cortices.

Taking advantage of the high temporal resolution of ERPs, Harris et al. (2013) demonstrated that the regulation of positive emotional reactivity involves both the early modulation of occipital cortex consistent with models of executive attention and perceptual filtering, and the late modulation of vmPFC activity relating to stimulus value. When viewed in the broader context, these findings have potentially far reaching implications for both our basic understanding of emotion regulation, and for furthering our understanding of how emotion regulation goes awry in disorders of emotion.

## Conflict of interest statement

The authors declare that the research was conducted in the absence of any commercial or financial relationships that could be construed as a potential conflict of interest.
